# Consumers' Knowledge, Attitudes, and Practices Toward Calorie Labeling in Riyadh City, Saudi Arabia: A Cross-Sectional Assessment

**DOI:** 10.3389/fpubh.2022.893978

**Published:** 2022-07-14

**Authors:** Nouf M. AlShehri, Mezna A. AlMarzooqi

**Affiliations:** Department of Community Health Sciences, College of Applied Medical Sciences, King Saud University Riyadh, Riyadh, Saudi Arabia

**Keywords:** menu calorie labeling, KAP study, barriers, Saudi Arabia, KAP (knowledge, attitude, and practice)

## Abstract

**Background:**

Menu calorie labeling is a useful means to encourage consumers to be informed about healthy eating and food choices. It is projected as an innovative method that will change the food environment and increases consumers' awareness of calories.

**Objective:**

This study aims to determine the consumer's knowledge, attitudes, and practices toward menu calorie labeling in Saudi Arabia.

**Methods:**

This is a descriptive cross-sectional study involving 435 consumers in Saudi Arabia. The participants filled out an online electronic survey questionnaire that assesses the demographic factors, knowledge, attitudes, practices, and barriers toward menu calorie labeling. Logistic regression was performed to determine the predictor of attitudes of consumers toward menu calorie labeling.

**Results:**

Of those 435 consumers, 50.1% were men, 33% were in the age group of 30–39, and 49.4% had a bachelor's degree. The majority of the participants reported that they can understand the calorie labels that were presented on the menus of the restaurants (*N* = 365, 83.9%). A high percentage of participants reported that calorie labeling encourages them to choose foods with a smaller number of calories (*N* = 387, 89%) and supported the posting of calorie content next to the price of the food items on the menus (*N* = 405, 93.1%). Barriers to using calorie labels were time-consuming and low-calorie food items are usually costly. Gender and educational attainment were found significantly associated with consumers' knowledge while marital status and BMI level were found significantly associated with attitudes and practices to using calorie labels (*p* < 0.05).

**Conclusion:**

Overall, the participants had adequate knowledge and positive attitudes about menu calorie labeling in Saudi Arabia. Menu calorie labeling may be a useful policy tool for promoting healthy eating habits and appropriate caloric consumption.

## Introduction

Nutritional information in restaurant menus aims to help consumers to make healthier dietary choices. In recent years, menu calorie labeling has been implemented worldwide particularly in fast-food restaurants to encourage consumers to choose fewer calorie foods. The consumption of high caloric food has been associated with obesity, type 2 diabetes mellitus, and cardiovascular disease (1, 2). Saudi Arabia is among the top countries in the Eastern Mediterranean region in the prevalence of obesity and diabetes (3, 4). For example, the prevalence of obesity has reached 35.6% of the Saudi population and it is expected to rise by 2030 (5, 6). The treatment and prevention of these non-communicable diseases nowadays are focused on psychological and social factors, particularly dietary management and behavioral change.

Several countries like the United States made a statute and implemented restaurant outlets to label nutrition information on their menus (7, 8). The US Food and Drug Administration in 2018 started requiring large food chain establishments to label their menus with calorie information for compliance with the menu labeling provision of the 2010 Affordable Care Act.3 (9). In Saudi Arabia, the Saudi Food and Drug Authority (SFDA) in 2017 introduced mandatory calorie labeling on the menus of the restaurants, bakeries, and cafes, as part of its vision of 2030, to improve eating habits among consumers (10). Menu calorie labeling is one of the suggested policies which can be defined as displaying the kilocalorie (calorie) content of food items listed on a menu (11). It is considered a useful means to encourage consumers to be informed about healthy eating and food choices. Menu calorie labeling is projected as an innovative method that will change the food environment, increases consumers' awareness of calories, and will help in prevention of obesity (12, 13). In addition, menu calorie labeling implementation policy has been linked to being cost-effective in both healthcare and society (14, 15). Because menu calorie labeling can support consumers' food choices and alter their dietary habits while restaurants are encouraged to reformulate their menus with healthier alternatives (14, 15).

Several studies have been done to investigate the effects of implementing a menu calorie labeling policy on energy consumption among consumers (16–18). Most of the previous studies, reported partially positive effects of calorie labeling implementation in the cafeterias, while others showed little or no effects, particularly in fast food restaurants (19–22). Other studies reported barriers to not using calorie labeling among the consumers, such as cost, time limits, and difficulties in understanding calorie meaning, along with the influence of hunger, preference, and ordering habits (23–26). Although the importance and the need for such research have been defined, little has been undertaken in Saudi Arabia on such an issue. Furthermore, assessing the effectiveness of this policy is important and as the consumers become more alert to the associations between dietary intake and illnesses, their level of awareness about the nutritional aspects of the calorie labeling should be increased. Also, identifying these barriers is essential for providing applicable and appropriate suggestions to food labeling makers about consumers' requirements in terms of food labels. Therefore, we sought to determine the consumer's knowledge, attitudes, and practices toward menu calorie labeling in Saudi Arabia and its association with their demographic characteristics. We also aimed to identify the barriers to not using calorie labeling among consumers in Saudi Arabia.

## Methods

### Study Design

This study was designed as a descriptive cross-sectional study.

### Sample and Setting

A convenience sample of 435 consumers in Riyadh, Saudi Arabia was surveyed to measure their knowledge, attitudes, and practices toward menu calorie labeling. The capital city of Saudi Arabia is Riyadh, and according to the General Authority of Statistics (https://www.stats.gov.sa/en), Riyadh has the second largest number of restaurants in the country. The eligibility criteria of this study included being an adult man or woman aged > 18 years old and a Saudi national. The sample size was calculated using an online sample size calculator (http://www.raosoft.com/samplesize.html). The appropriate sample size was 385 participants based on a 5% margin of error, a confidence interval of 95%, and the current population in Riyadh, Saudi Arabia. A 20% margin of error was added to the sample for clustering effect and missing data to ensure and reach the required number of samples.

### Instrument

All consented participants answered a validated questionnaire designed from the previous study conducted in England (26). Additional questions were added to assess the possible barriers to not using the calorie labels among consumers derived from previous studies in the United States, and the United Kingdom (23–26). The questionnaire consisted of 5 sections, the first section was the demographic characteristics, which included gender, age, educational level, marital status, household income, and self-reported questions contained anthropometric measurements: weight in kilograms (kg) and height in centimeters (cm).

The second section includes 6 factual statements that were designed to assess the participants' awareness and knowledge of calorie labeling policy and nutritional information regarding the calories' meanings and calculations. All participants responded with a “yes” or “no,” with one point was given for each correct answer. The third section was designed to ask about the participant's level of agreement regarding some attitudes toward calorie labeling. The fourth section focused on the practices of calorie labeling, and participants were asked to choose their answers based on the frequency level of each practice given. The last section was designed to assess the barriers to not using the calorie labels among consumers. A scoring system was applied in which questions related to attitudes, practices, and barriers to not using calorie labels when eating or purchasing food items were measured using a 5-point Likert scale ranging from 1 = strongly disagree to 5 = strongly agree. An overall summation score was computed for the entire scale in each section, on which higher scores indicate a positive attitude toward calorie labeling and an extreme concern in each statement. To further strengthen the reliability of this research and ensure that the adaptation was culturally appropriate, a pilot study was administered. A forward-translation and back-translation method were performed by a professional Arabic-English translator to those consumers who were willing to participate in the study. The Arabic version of the questionnaire has high reliability with reported Cronbach's alpha coefficients of.72 (knowledge), 0.71 (attitudes), 0.84 (practices), and 0.76 (barriers).

### Data Collection Procedure

The researcher created an online survey distributed through social media (e.g., WhatsApp and Twitter). The data was collected from January 2020 to April 2020 from different public consumers in Saudi Arabia. The participants were recruited from different groups recommended by professors and physicians of the clinical nutrition department of King Saud University. According to the professors and physicians, the participants were part of the health promotion activities of the department from different areas in Saudi Arabia. Prior to distributing the survey questionnaire, the participants were informed about the aim of the research, about the confidentiality of their participation, and about their right to withdraw from the study anytime. Also, the researchers informed that they have the option to refuse or discontinue answering the study.

### Statistical Analysis

The data were statistically analyzed using SPSS, version 24 statistical program (SPSS Inc., Chicago, IL, USA). Descriptive analysis, frequencies, and percentages were calculated for the demographic characteristics. The Chi-square test was used to examine the correlation between any two variables. Logistic regression was performed to assess the independent relationship between the predictor variables and attitudes of consumers toward menu calorie labeling. Statistical significance was considered at a *P-*value < 0.05 for all analyses.

### Ethics Approval and Consent to Participate

The Institutional Review Board Committee at King Saud University approved the study prior to enrollment of participants in this study. (Approval no: E1-21-691–Reference no: 21/0707/IRB).

## Results

The demographic characteristics of the participants are shown in Table 1. As reflected, the total sample was 435 participants with almost similar and equal percentages of men (*N* = 218, 50.1%) and women (*N* = 217, 49.9%). A total of 30% of the participants were in the age group of 30–39 years old, 25.5% of 40–49 years old, and 23.2% of 18–29 years old. Only 17% of the respondents were aged 50 and above. Among the participants, 49.4% had a bachelor's degree, 14% completed a master's or doctoral degree, and 6% had at least less than high school education. In terms of family income, thirty-eight percent of the participants had a family income of 10,000–19,999 SR. Based on the respondent's BMI scores, nearly half of the study's participants were classified as overweight and obese (39.8 and 36.1%, respectively), while only 21.6% of all participants showed normal BMI.

**Table 1:** Demographic characteristic of the participants.

**Variable**	***N* = 435**	**%**
**Age**		
18–29 years	101	23.2
30–39 years	145	33.3
40–49 years	111	25.5
50 and above	78	17.9
**Gender**		
Men	218	50.1
Women	217	49.9
**Marital status**		
Single	123	28.3
Married	312	71.7
**Educational level**		
Less than high school	27	6.2
High school	75	17.2
Diploma	57	13.1
Bachelor's degree	215	49.4
Masters or postgraduate degree	62	14
**Income**		
Less than 10000 SR	103	23.7
10000–19,999 SR	169	38.9
20, 000–29,999	82	18.9
More than 30,000	81	18.6
**BMI Level (kg/m** ^**2**^ **)**		
Underweight (<18.5 kg/m^2^)	11	2.5%
Normal (18.5–24.9 kg/m^2^)	94	21.6%
Overweight (25–29.9 kg/m^2^)	173	39.8%
Obesity (≥ 30 kg/m^2^)	157	36.1%

Table 2 describes the knowledge of the respondents about menu calorie labeling. Overall, the majority of the participants had adequate knowledge about menu calorie labeling, particularly about the meaning of calories. A high majority of the participants (83.9%) reported that they can understand the calorie labels that were presented on the menus of the restaurants. More than half of the participants reported that they are familiar with the menu calorie labeling policy in Saudi Arabia (*N* = 293, 67.4%). However, only or nearly half of the participants know their calorie requirements (*N* = 222, 51%), were able to calculate the calorie content of some food items (*N* = 215, 49.4%), and were able to calculate their energy intake during the day (*N* = 189, 43.4%).

**Table 2:** Assessment of knowledge, attitudes, and practices of participants toward menu calorie labeling.

	**Variables (*N* = 435)**	**Yes, know enough**		**No, do not know enough**	
	**Knowledge toward menu calorie labeling**	**N**	**%**	**N**	**%**
1	I know what is the meaning of calories	406	93.3	29	6.7
2	I know my calorie requirements	222	51.0	213	49.0
3	I am familiar with the menu calorie labeling policy	293	67.4	142	32.6
4	I can understand the calorie labels on the menus	365	83.9	70	16.1
5	I know how to calculate the calories content of food items	215	49.4	220	50.6
6	I can calculate my calories intake during the day	189	43.4	246	56.6
	***Attitudes toward menu calorie labeling 435***	**Strongly agree–agree**		**Neither**		**Strongly disagree–disagree**		**Mean (SD)**
7	I think that posting the calorie information for each food item in the menu is useful.	397	91.3	30	6.9	8	1.8	4.46 (0.72)
8	I find it useful to post the calorie count for each food item in the menu	410	94.3	18	4.1	7	1.6	4.58 (0.66)
9	Posting calorie information in the menu board would encourage me to select food with less calorie.	387	89.0	32	7.3	16	3.7	4.47 (0.82)
10	I feel guilty for picking a higher calorie food if calories were posted.	326	74.9	63	14.5	46	10.6	4.10 (1.09)
11	I am with the government requiring the restaurants to post calorie information on menu boards for each food item.	413	94.9	16	3.7	6	1.4	4.70 (0.63)
12	I am with posting the calories information next to the price of the food items in the menus	405	93.1	18	4.1	12	2.8	4.58 (0.76)
13	I overestimate the menu calorie labels condition	194	44.6	170	39.1	71	16.3	3.42 (0.97)
14	I underestimate the menu calorie labels condition	193	44.4	170	39	72	16.6	3.41 (0.98)
	**Practices toward calorie labeling**	**Always–usually**		**Neither**		**Rarely–never**		
15	I am eating away from home.	100	22.9	226	52.1	109	25.0	3.00 (0.79)
16	I prefer to eat at restaurants that post the calorie information on the menu.	214	49.1	130	30	91	20.9	3.37 (1.20)
17	I pay attention to the calorie labels when choosing my order.	111	25.5	242	55.7	82	18.8	3.53 (1.17)
18	I choose foods with a smaller number of calories.	227	52.1	124	28.6	84	19.3	3.36 (1.10)
19	I avoid foods posted with high calorie content.	195	44.8	168	38.7	72	16.5	3.44 (1.10)
20	I look at the calorie labels when purchasing foods from the markets.	182	41.9	112	25.7	141	32.4	3.20 (1.29)
21	I calculate my total energy intake during the day	111	25.5	102	23.5	222	51.0	2.63 (1.28)
	***Barriers toward menu calorie labeling***	**Strongly agree–agree**		**Neither**		**Strongly disagree–disagree**		
22	I am not interested to know the calorie content of the food anyway. ^*^	69	15.9	99	22.5	267	61.4	3.66 (1.07)
23	I don't care about the calorie information of the food items that I love to eat. ^*^	97	22.3	83	19.1	255	58.6	3.50 (1.15)
24	I find it difficult to understand the calorie content of the food items posted on the menus.	110	25.3	85	19.5	240	55.2	2.59 (1.19)
25	It takes time to read the calorie labels of the food items on the menus.	196	45.1	86	19.7	153	35.2	3.09 (1.14)
26	I don't care about the calorie content of the food items when I am feeling hungry.	201	46.2	88	20.2	146	33.6	3.16 (1.23)
27	The low-calorie food items are usually costly.	240	55.2	128	29.4	67	15.4	3.60 (1.19)

* *Negative attitude statements were scored from 1 (for those who strongly agreed) to 5 (for those who strongly disagreed)*.

Attitudes of the participants toward menu calorie labeling are also presented in Table 2. The majority of the participants agreed on the usefulness of calorie labeling (*N* = 410, 94.3%) and with the government for requiring the restaurants to post calorie information on menu boards for each food item (*N* = 413, 94.9%). A high percentage of participants reported that calorie labeling encourages them to choose foods with a smaller number of calories (*N* = 387, 89%) and supported the posting of calories content next to the price of the food items on the menus (*N* = 405, 93.1%). Meanwhile, nearly half of the respondents think that posting calories on the menus were overestimated (*N* = 194, 44.6%), while a similar percentage think it was underestimated (*N* = 193, 44.4). Nearly half of the participants preferred to eat at restaurants that post the calorie information on the menu (*N* = 214, 49.1%) and avoid food that is posted with high calorie content (*N* = 195, 44.8%). A small percentage of the participants pay attention to calorie labels when choosing their order (*N* = 111, 25.5%) and calculate their total energy intake during the day (*N* = 111, 25.5%). Moreover, 52.1% (*N* = 227) of the participants choose foods with a smaller number of calories. A total of 25% of the participants reported rarely eating at home while 41.9% (*N* = 182) of the participants look at calorie labels when purchasing foods from the markets.

The barriers influencing non-use of menu calorie labeling are presented in Table 2. More than half of the participants were interested to know the calorie content of the food (*N* = 267, 61.4%) and cared about the calorie content of the food they love to eat (*N* = 255, 58.6%). Moreover, 55.2% (*N* = 240) of the participants have found it easy to understand the calorie content posted on the menus. Meanwhile, nearly half of the participants reported that it takes time to read the calorie labeling on the menu (*N* = 196, 45.1%) and do not care about the calorie content of the food items when they are hungry (*N* = 201, 46.2%). Interestingly, more than half of the participants reported that low-calorie food items are usually costly (*N* = 240, 55.2%).

A chi-square analysis was employed to identify the association between consumers' knowledge, attitudes, and practices toward menu calorie labeling with their demographic characteristics (Table 3). Of the 6 factors, two (gender and educational attainment) were found to be significantly associated with consumers' knowledge (*P* < 0.05). The attitudes toward menu care labeling and consumers' marital status and BMI level were found to be significantly associated (*p* < 0.05). Significant associations between educational level and BMI level were reported for these practices (*P* < 0.05). The analysis also proves that there is a statistically significant association between consumers' BMI levels and barriers to using calorie labels when eating or purchasing food items. No significant association was found with other demographic characteristics.

**Table 3:** Association between participants' knowledge, attitudes, and practices with demographic characteristics.

**Variable**												
**Age**	**Knowledge**			**Attitude**			**Practice**			**Barriers**		
	**High**	**Low**	***p*-value**	**Agree**	**Disagree**	***p*-value**	**Always-usually**	**Rarely-never**	***p-*value**	**Strongly agree-agree**	**Strong disagree-disagree**	***p*-value**
18–29 years	89 (88.1)	12 (11.9)	0.335	92 (22.4)	0	0.092	44 (43.5)	21 (20.7)	0.259	15 (18.5)	38 (21.5)	0.181
30–39 years	124 (85.5)	21 (14.5)		137 (33.4)	3 (2.0)		76 (52.4)	27 (18.6)		23 (28.4)	63 (35.6)	
40–49 years	90 (81.1)	21 (18.9)		105 (25.6)	0		48 (43.2)	27 (24.3)		22 (27.2)	42 (23.7)	
50 and above	62 (79.5)	16 (20.5)		76 (18.5)	0		34 (43.5)	22 (28.2)		21 (25.9)	34 (19.2)	
**Gender**			**0.004**			0.156			0.086			**0.050**
Men	113 (44.3)	105 (58.3)		206 (50.2)	3 (1.3)		95 (43.5)	59 (28.2)		63 (28.8)	110 (50.4)	
Women	142 (55.7)	75 (41.7)		204 (49.8)	0		107 (49.3)	38 (17.5)		47 (21.6)	130 (59.9)	
**Marital status**			0.105			**0.046**			0.407			0.761
Single	108 (87.8)	15 (12.2)		102 (82.9)	9 (7.3)		59 (47.9)	24 (19.5)		21 (17.0)	49 (39.8)	
Married	257 (82.4)	55 (17.6)		285 (91.3)	7 (2.24)		133 (42.6)	73 (23.3)		60 (19.2)	128 (41.0)	
**Educational level**			**0.019**			0.167			**0.022**			0.535
Less than High school	15 (5.9)	12 (6.7)		27 (100)	0		14 (51.8)	2 (7.4)		7 (25.9)	9 (33.3)	
High school	42 (16.5)	33 (18.3)		68 (90.6)	2		32 (42.6)	14 (18.6)		18 (24)	29 (38.6)	
University or college	157 (57.7)	115 (42.2)		266 (97.7)	0		132(48.5)	66 (24.2)		48 (17.6)	110 (40.4)	
equivalent											
Postgraduate degree	41 (66.1)	20 (37.2)		59 (95.1)	1 (1.6)		24 (38.7)	15 (24.1)		8 (12.9)	29 (46.7)	
**Income**			0.702			0.845			0.100			0.473
Less than 10000 SR	84 (81.6)	19 (18.4)		92 (89.3)	5 (4.8)		15 (14.5)	1 (0.9)		6 (5.8)	11 (10.6)	
10000–19,999 SR	138 (81.7)	31 (18.3)		148 (87.5)	6 (3.5)		37 (21.8)	16 (9.4)		16 (9.4)	27 (15.9)	
20, 000–29,999	72 (87.8)	10 (12.2)		76 (92.6)	3 (3.6)		69 (84)	46 (4.8)		35 (42.6)	62 (7.3)	
More than 30,000	71 (87.1)	10 (12.3)		71 (47.7)	2 (2.4)		41 (50.6)	14 (17.2)		11 (13.5)	39 (48.1)	
**BMI Level (kg/m** ^**2**^ **)**			0.054			**0.001**			**0.007**			**0.034**
Underweight (<18.5 kg/m^2^)	6 (2.4)	5 (2.8)		7 (1.7)	0		5 (45.1)	2 (18.1)		5 (45.1)	5 (45.1)	
Normal (18.5–24.9 kg/m^2^)	64 (25.1)	30 (16.7)		85 (20.7)	2 (66.7)		20 (21.2)	47 (50)		26 (27.6)	59 (62.7)	
Overweight (25–29.9 kg/m^2^)	105 (41.2)	68 (37.9)		164 (40.0)	1 (33.3)		33 (19.0)	92 (53.1)		58 (33.5)	95 (54.9)	
Obese (≥ 30 kg/m^2^)	80 (31.4)	77 (42.8)		154 (37.6)	0		42 (27.0)	61 (39.2)		65 (41.9)	74 (47.7)	

*Missing sample/numbers were in neither/neutral; p-value significant at < 0.05 level*.

*The bold values indicate statistical significance (p < 0.05)*.

Logistic regression was performed to assess the impact of a number of factors on the likelihood that participants would report attitudes toward menu calorie labeling. The model contained five independent variables (age, gender marital status, educational level, monthly income, and BMI level). As shown in Table 4, only two independent variables emerged with a unique statistically significant contribution to the model (educational level and BMI level). The strongest predictor of attitudes in menu calorie labeling was BMI level, recording an odds ratio of 1.17. This indicated that participants who were overweight and obese were one time more likely to have a positive attitude toward menu care labeling (*p* = 0.044).

**Table 4:** Logistic regression predicting Likelihood toward attitudes in menu calorie labeling.

**Variable**	
**Age**	**Odds ratio (95% confidence interval)**	**SE**	***p*-value**
18–29 years	1	0.01	0.728
30–39 years	0.18 (0.02–0.67)		
40–49 years	0.11 (0.08–1.42)		
50 and above	0.07 (0.02–1.28)		
**Gender**			
Men	1	0.686	0.762
Women	1.23 (0.32–4.71)		
**Marital status**			
Single	1		
Married	0.61 (0.14–2.69)	0.75	0.519
**Educational level**			
Less than High school - High school	1	0.415	**0.050**
University or college equivalent	0.12 (0.12–1.27)		
Postgraduate degree	0.56 (0.06–5.15)		
**Income**			
Less than 10000 SR	1	0.958	0.555
10000–19,999 SR	0.30 (0.04–1.96)		
20, 000–29,999	0.59 (0.10–3.46)		
More than 30,000	0.32 (0.46–2.32)		
**BMI Level (kg/m** ^**2**^ **)**			
Underweight (<18.5 kg/m^2^)	1	1.17	**0.044**
Normal (18.5–24.9 kg/m^2^)	0.10 (0.03–1.03)		
Overweight (25–29.9 kg/m^2^)	0.23 (0.04–1.36)		
Obese (≥ 30 kg/m^2^)	0.66 (0.11–3.94)		

*CI, Confidence Interval; SE, Standard Error; p-value significant at < 0.05 level*.

*The bold values indicate statistical significance (p < 0.05)*.

## Discussion

This study investigated the consumer's knowledge, attitudes, and practices toward menu calorie labeling in Saudi Arabia and its association with their demographic characteristics. Barriers and predictors of the attitudes toward menu calorie labeling were identified based on the self-reported responses of the participants.

As reported, the majority of the participants had adequate knowledge about menu care labeling particularly the meaning of calories, menu calorie labels, and calorie label policy in Saudi Arabia. However, nearly or only half of the participants do not have knowledge about their calorie requirements and how to calculate their energy intake during the day. The finding of this study is parallel with the study done in Canada and the United States (27, 28). Educational efforts to increase knowledge and use of calorie information may be helpful to improve the awareness and promote healthy food consumption of consumers in Saudi Arabia. Meanwhile, the respondents of this study have expressed positive attitudes and support toward the menu calorie labeling policy and its usefulness. The attitudes and support of the population toward menu calorie labeling are essential in the implementation and success of this policy. According to Krieger et al., garnering positive attitudes from consumers impacted the effectiveness of, and necessitated, the menu labeling policy (29). With regards to practices toward menu care labeling, the study shows that nearly half of participants preferred to eat at restaurants that post the calorie information on the menu. This is in accordance with the findings of a cross-sectional study among 196 Saudi adolescents that revealed nearly half of the adolescents consumed fast-food one time per week and that 20% consumed more than two times a week (30). Another study has shown high consumption of high-fat fast food among adolescents in Saudi Arabia (31). Meanwhile, a study in Jeddah revealed a high prevalence of junk/fast food consumption among Saudi adults (32). The frequency of fast-food items intake among adolescents and adults ranged from one to 3 times per week, which could significantly expose them to different conditions detrimental to their health. Fast food items are rich in salt, and excess sodium can adversely target blood vessels and organs such as the heart and kidneys (33).

Interestingly, a small percentage of the participants pay attention to calorie labels when choosing their order. This result is similar to a study by Block (2013) that reported consumers to underestimate the nutritional content like sodium, sugar, fat, and calorie of items when eating outside or in restaurants (34). Previous studies have noted that eating in restaurants is associated with lower micronutrients, weight gain, and increased body fat than eating at home (35, 36). Health education programs may be needed to increase consumer awareness of the nutritional values and content of each item on the menu, which will promote eating healthier foods.

Another highlight of this study is the barriers to using calorie labels among consumers. Similar findings were reported from previous studies (23–26). With more countries implementing menu calorie labeling, barriers arise such as the influence of hunger, preference, cost, time limits, difficulties in the understanding of calorie meaning, and ordering habits, all of which are considered barriers to menu label usage. Implementation of menu calorie labeling is a complex process and needs to be explored and reviewed. Future studies should assess different display methods considering the demographic differences among the consumers in Saudi Arabia.

Interestingly, our study also revealed that there was a statistically significant association between consumers' knowledge, attitudes, and practices toward menu calorie labeling with their demographic characteristics. The findings of this study were parallel with those studies conducted in Iran and the United Arab Emirates (37, 38). Besides, a statistically significant association was found between the subject's knowledge and their education level (*P* = 0.019). For most of the participants who had good knowledge about calorie labeling, their educational level was a “Bachelor's degree and more,” which means that the higher their educational level, the higher their knowledge of calorie labeling. In other studies, similar associations were reported (8, 39, 40). These results could be affected by the type of population, for example, participants working in a hospital cafeteria are known to be more educated because they are more frequently exposed to the calorie labeling intervention than participants in public areas such as restaurants. Therefore, increasing the awareness among less-educated consumers is very important to assure the beneficial outcomes of calorie labeling, and more educational programs should be established, starting from the schools, to increase the level of awareness regarding healthy food consumption.

Moreover, statistically significant associations were found between the participant's attitudes and practices with their BMI level. This is in accordance with a study in India that high BMI was significantly associated with fast food consumption (41). Calorie labeling in fast food may help or lead consumers to select lower calorie items or fewer items on the menu. Also, a statistically significant association was found between the subject's practices toward calorie labeling and their education level, which emphasizes that the higher educated participants have better practices toward calorie labeling. Similarly, similar findings were reported in other studies (13, 34, 36). A previous study highlighted that, among college students, their weight status and weight concerns were predictive of changes in calories in what they ordered in a fast food restaurant (42). This is in line with our finding in which educational attainment and BMI level were found as significant predictors of attitudes toward menu calorie labeling.

The study has some limitations. First, the study design is cross-sectional that only addresses associations and cannot detect causal relationships. Second, some questions cannot be answered with a simple 'yes' or 'no,' which would require the participants to elaborate on their views. Further studies are needed to elaborate on the responses on knowledge, attitudes, and barriers to menu calorie labeling. Lastly, the results of this study cannot be generalized to the whole population in Saudi Arabia. However, the present study's findings are of value to food-labeling makers to the recommendations that have been suggested for improving calorie labeling policy in the country.

Overall, the participants had adequate knowledge and positive attitudes about menu calorie labeling in Saudi Arabia. This study also identified barriers to using calorie labels among the consumers such as time-consuming and low calorie food items are usually costly. Menu calorie labeling may be a useful policy tool for promoting healthy eating habits and appropriate caloric consumption. However, most of the studies that have been done to assess the outcomes of calorie labeling policy reported partially positive effects in the cafeterias, while others showed little or no effects, particularly in fast food restaurants. A user-friendly way of promoting and presenting nutrition information may increase the consumers' knowledge about dietary reference values. In addition, to improve the effectiveness of this policy and encourage consumers to make healthier choices and manage their caloric intake, more studies are needed to evaluate the reliability of the current calorie labels that were implemented in the food settings of Saudi Arabia. Future studies should assess different display methods considering the demographic differences among the consumers in Saudi Arabia. Health education programs may help increase awareness and encourage consumers to choose and practice eating healthier foods.

## Data Availability Statement

The data that support the findings of this study are available at the Department of Community Health Sciences, College of Applied Medical Science King Saud University, but restrictions apply to the availability of these data, which were used under license for the current study, and so are not publicly available. Data are however available from the authors upon reasonable request.

## Ethics Statement

The studies involving human participants were reviewed and approved by the Institutional Review Board Committee at King Saud University approved the study prior to enrollment in this study (Approval no: E1-21-691–Reference no: 21/0707/IRB). The patients/participants provided their written informed consent to participate in this study.

## Author Contributions

NA and MA contributed to data analysis, interpretation of results, drafting or revising the manuscript, and agree to be accountable for all aspects of the work. Both authors have contributed to and approved the final version of the manuscript.

## Funding

This research project was supported by a grant from the Research Center of the Female Scientific and Medical Colleges, Deanship of Scientific Research, King Saud University.

## Conflict of Interest

The authors declare that the research was conducted in the absence of any commercial or financial relationships that could be construed as a potential conflict of interest.

## Publisher's Note

All claims expressed in this article are solely those of the authors and do not necessarily represent those of their affiliated organizations, or those of the publisher, the editors and the reviewers. Any product that may be evaluated in this article, or claim that may be made by its manufacturer, is not guaranteed or endorsed by the publisher.
